# Surgery and adjuvant dendritic cell-based tumour vaccination for patients with relapsed malignant glioma, a feasibility study

**DOI:** 10.1038/sj.bjc.6602195

**Published:** 2004-10-12

**Authors:** S Rutkowski, S De Vleeschouwer, E Kaempgen, J E A Wolff, J Kühl, P Demaerel, M Warmuth-Metz, P Flamen, F Van Calenbergh, C Plets, N Sörensen, A Opitz, S W Van Gool

**Affiliations:** 1 Department of Pediatric Oncology, Children's Hospital, University of Wuerzburg, Josef-Schneider-Str. 2, D-97080 Wuerzburg, Germany; 2 Laboratory of Experimental Immunology, University Hospital Gasthuisberg, Herestraat 49, B-3000 Leuven, Belgium; 3 Department of Neurosurgery, University Hospital Gasthuisberg, Herestraat 49, B-3000 Leuven, Belgium; 4 Department of Dermatology, University of Erlangen, Hartmennstrasse 14, D-91052 Erlangen, Germany; 5 Department of Pediatric Oncology, St Hedwig, University of Regensburg, Steinmetzstr. 1-3, D-93049 Regensburg, Germany; 6 Department of Radiology, University Hospital Gasthuisberg, Leuven, Herestraat 49, B-3000 Leuven, Belgium; 7 Department of Neuroradiology, University of Wuerzburg, Josef-Schneider-Str. 11, D-97080 Wuerzburg, Germany; 8 Department of Nuclear Medicine, Jules Bordet Institute, Héger-Bordetstraat 1, B-1000 Brussel, Belgium; 9 Department of Pediatric Neurosurgery, University of Wuerzburg, Josef-Schneider-Str. 11, D-97080 Wuerzburg, Germany; 10 Department of Transfusion Medicine, University of Wuerzburg, Josef-Schneider-Str. 2, D-97080 Wuerzburg, Germany; 11 Department of Pediatrics, University Hospital Gasthuisberg, Herestraat 49, B-3000 Leuven, Belgium

**Keywords:** glioma, immunotherapy, tumour vaccination, dendritic cells, adjuvant therapy

## Abstract

Patients with relapsed malignant glioma have a poor prognosis. We developed a strategy of vaccination using autologous mature dendritic cells loaded with autologous tumour homogenate. In total, 12 patients with a median age of 36 years (range: 11–78) were treated. All had relapsing malignant glioma. After surgery, vaccines were given at weeks 1 and 3, and later every 4 weeks. A median of 5 (range: 2–7) vaccines was given. There were no serious adverse events except in one patient with gross residual tumour prior to vaccination, who repetitively developed vaccine-related peritumoral oedema. Minor toxicities were recorded in four out of 12 patients. In six patients with postoperative residual tumour, vaccination induced one stable disease during 8 weeks, and one partial response. Two of six patients with complete resection are in CCR for 3 years. Tumour vaccination for patients with relapsed malignant glioma is feasible and likely beneficial for patients with minimal residual tumour burden.

In spite of modern oncological treatment, the prognosis of glioblastoma multiforme (GBM) remains dismal, with a median survival of less than 2 years (Young *et al*, 1981; Shrieve *et al*, 1999; Wolff *et al*, 2000). The prognosis at time of relapse is even worse (Finlay *et al*, 1995; Nieder *et al*, 2000; Brada *et al*, 2001; Tamber and Rutka, 2003; Rich *et al*, 2004). However, new treatment strategies are under development, one of them being immune therapy.

Brain tumours are considered to be located in a site of relative immune privilege (Walker *et al*, 2002). Malignant gliomas have immune suppressive characteristics locally (Black *et al*, 1992) and systemically (Elliott *et al*, 1990). In case of vaccination, immune responses are induced at sites remote from the tumour. Effector cells then recirculate to mediate their antitumour effects in the brain. The concept of tumour vaccination using dendritic cells (DC) has been demonstrated in animal models (De Vleeschouwer *et al*, in press). Phase I studies have demonstrated feasibility, safety and bioactivity of autologous peptide-pulsed DC vaccine for patients with malignant glioma (Yu *et al*, 2001). Early clinical experiences with immunotherapy using protein-pulsed DC suggest this to be a promising strategy for patients with recurrent malignant glioma (Wheeler *et al*, 2003; Yamanaka *et al*, 2003; De Vleeschouwer *et al*, 2004).

We summarise our experience in a group of patients with relapsed malignant glioma, who were treated with autologous DC loaded with proteins derived from autologous tumour homogenate.

## MATERIALS AND METHODS

### Patient population

All patients had a relapsing malignant glioma. Patients were considered candidates for adjuvant vaccination if a new operation with the intent to extensively debulk the tumour was deemed safe by the neurosurgeon. No further restrictions were applied to recruit the patients. In this feasibility study, no patient dropped out after reoperation. Patient characteristics are described in Table 1

**Table 1:** Patient characteristics

**Patient number**	**Age (year)**	**Sex**	**Time of prior events**	**Prior histology**	**Prior treatment**	**Time of last relapse**	**Histology at relapse**
1	60	M	02-2000	GBM	S, R, C	10-2000	GBM
2	11	F	02-1997	AA	S, R, C	05-2001	PXA
			05-1999	AA			
3	17	F	01-1989	PA	S, R	06-2001	GBM
			03-1999	AA	S, R, C		
4	15	M	02-1998	GBM	S, R, C	06-2001	GBM
5	34	F	07-2001	AA	S, R	07-2001	GBM
6	53	M	02-2001	GBM	S, R, C	12-2001	GBM
7	78	M	05-1999	GBM	S, R, C	02-2002	GBM
8	62	F	08-2001	GBM	S, R, C	04-2002	GBM
9	39	F	01-1995	ODG III	S, R, C	05-2002	GBM
			03-2000	ODG IV			
10	30	M	09-1999	AA	S, R, C	05-2002	GBM
11	15	F	12-1990	ALL	C	06-2002	No vital tissue after tumour bleeding
			10-2001	GBM	S, R		
12	62	F	4-9-2001	GBM	S, R, C	09-2002	GBM

AA=anaplastic astrocytoma; ALL=acute lymphatic leukaemia; C=chemotherapy; F=female; GBM=glioblastoma multiforme; III=WHO grade 3r, IV=WHO grade 4r; M=male; ODG=oligodendroglioma; PA=pilocytic astrocytoma; PXA=malignant pleomorphic xanthoastrocytoma; R=radiotherapy; S=surgery.

. There were 12 patients (seven female and five male) with a median age of 32 years (range: 11–78 years). Eight patients were vaccinated at first relapse, while four patients had more than one malignant event prior to vaccination. All patients were reoperated upon and were off steroids and nonsteroidal anti-inflammatory drugs at the time of vaccination. Approval by the local ethical committee was obtained, and informed consent was provided before the start of the immunotherapy.

### Assessment of extent of tumour resection before vaccination

Complete resection was defined as the absence of any residual tumour mass on early postoperative MRI or CT scan performed with and without contrast within 72 h after surgery. Any resection leaving a measurable residual tumoral mass <1 cm^3^ and <10% of the initial tumour volume was considered subtotal. All solid residual tumour of a measurable size ⩾1 cm^3^ or removal of <90% of the tumour volume was classified as partial resection.

### Tumour homogenate

Tumour tissue was immediately transported from the operation room into the laboratory and snap-frozen in liquid N_2_ without additives. For further preparation, the tissue was thawed and put into NaCl 0.9% with 1% human serum albumin, and was homogenised mechanically. Afterwards, six snap freeze/thaw cycles were performed. The homogenate was filtered with a Falcon filter (70 *μ*m). The amount of protein was measured using the Coomassie blue staining method and spectrophotometry at 595 nm (Bradford, 1976). After irradiation (60 Gy), the homogenate was kept frozen in liquid nitrogen until use.

### Preparation of autologous DC

In eight patients peripheral blood mononuclear cells (PBMC) were isolated from fresh blood samples. DC were differentiated out of the monocytes in the presence of 20 ng ml^−1^ rIL-4 (Pepro Tech Inc., Rocky Hill, NJ, USA) and 1000 U ml^−1^ rGM-CSF (Leukomax®, Novartis) for 7 days as described (Sallusto and Lanzavecchia, 1994). In the other patients, PBMC were obtained from leukapheresis, and kept frozen until use. For each vaccination, part of the PBMC was thawed, and adherent cells were differentiated to immature DC as described (Thurner *et al*, 1999). Immature DC were loaded with 30–200 *μ*g of tumour proteins per million DC, depending on the material available. For the loading procedure, 0.01% autologous plasma was used during the first 2 h, 0.04% for the next 4 h, and finally 0.28% for the last 20 h. At time of loading, rTNF-*α* (Strathmann Biotec AG, Dengelsberg, Germany), rIL-1*β* (Strathmann Biotec AG) and PGE2 (Prostin®, Pharmacia) were added in a final concentration of 120, 120 and 20 *μ*g ml^−1^ respectively. After 24 h, mature loaded DC were resuspended in PBS with 0.5% human serum albumin (HSA) at a concentration of 2–6 × 10^6^ ml^−1^. The syringes contained 1–2 million mature DC.

### Vaccination

Vaccination was performed by intradermal (i.d.) injection of 2–4 million DC per lymph node region in the upper third of the arms at weeks 1, 3, and further with an interval of 4 weeks. The patients were kept in the hospital for 2 h after vaccination.

### Skin tests

Delayed type hypersensitivity reaction (DTH) was tested after at least two vaccinations. For this, 100 *μ*l tumour homogenate and 100 *μ*l control PBS/HSA were injected i.d. After 24, 48, and 72 h, redness and induration were assessed by an independent observer. DTH reactions were judged as positive if the average perpendicular measurement of the reaction exceeded 5 mm.

### Patient assessment

All patients were followed by clinical examination and MRI scanning. In eight patients, methionine-PET imaging was performed. Imaging studies were scheduled before each vaccine except the second vaccine. Afterwards, clinical examination and imaging studies were performed every 3–4 months.

## RESULTS

### Vaccines preparation and characterisation

The patients received 2–7 (median: 5) vaccines after surgery. The details of the vaccinations for each patient are described in Table 2

**Table 2:** Vaccination data

**Patient number**	**Surgery prior to vaccination**	**Source of PBMC**	**Number of vaccinations**	**Amount of cells injected per vaccination (× 10^6^)**	**Skin test**
1	Partial resection	Leukapheresis	5	5/18/17/16/15	Not done
2	Total resection	Leukapheresis	7	4/3/4/16/10/14/13	−V2, +V6
3	Total resection	Fresh blood	6	3/1/2/9/1/2	+V3
4	Total resection	Leukapheresis	4	13/11/10/9	−V3
5	Total resection	Fresh blood	5	3/1.4/4.8/3/5	Not done
6	Total resection, second localisation	Fresh blood	3	8/7/6	+V3
7	Subtotal resection	Fresh blood	5	12/2/7/12/9	+V3
8	Partial resection	Fresh blood	2	13/6	Not done
9	Subtotal resection	Fresh blood	5	9/3/2.4/2.5/0.8	−V3
10	Subtotal resection	Fresh blood	5	4.8/2.1/1.4/5.5/6	+V3
11	Total resection (no vital tumour)	Leukapheresis	6	15/8/6/4/4/4	Not done
12	Total resection	Fresh blood	6	10/13/9/5/4.2/3	+V3

Vx=vaccine number.

**Figure 1:**
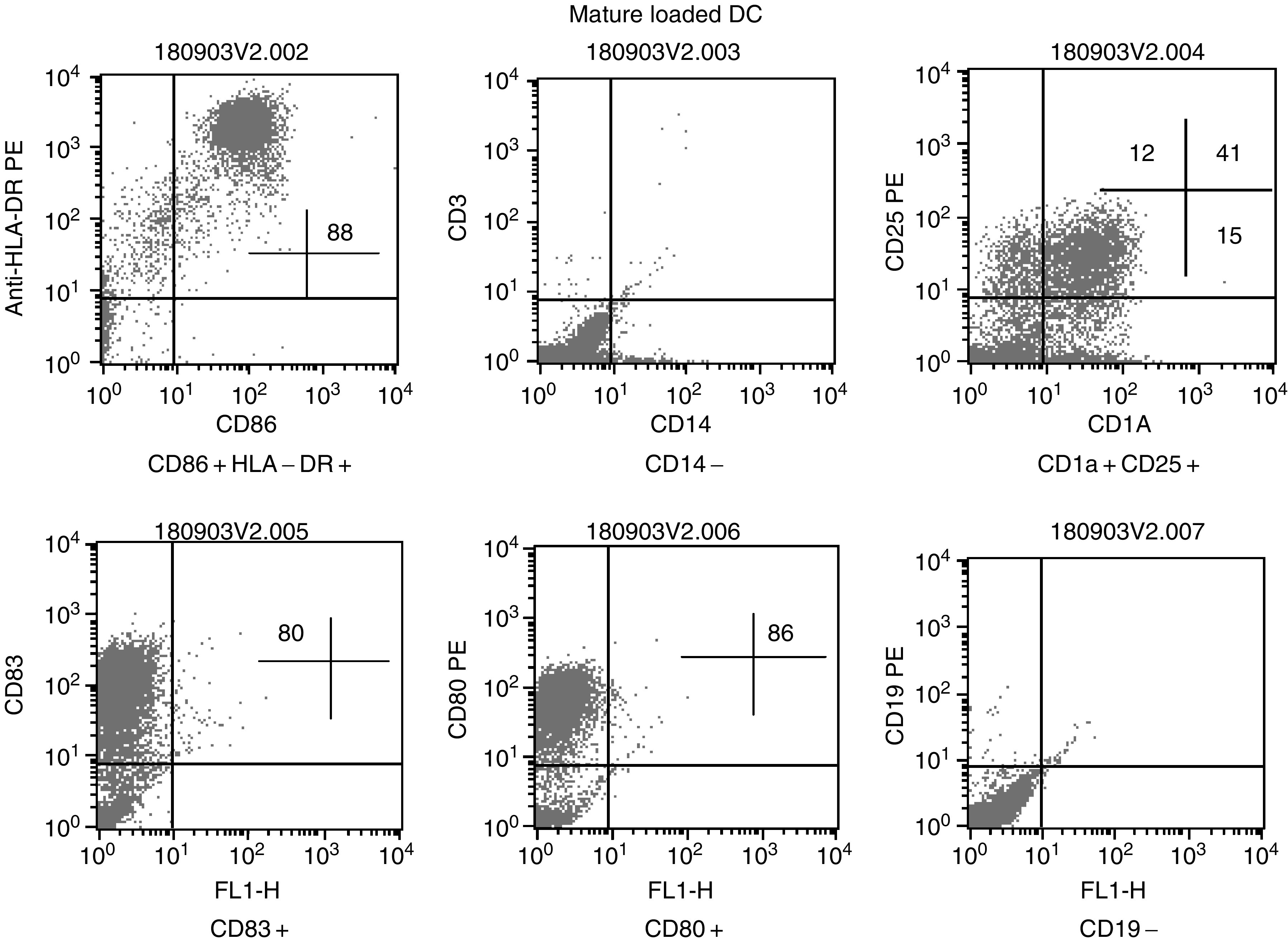
Quality control of dendritic cells. Representative example obtained by FACS analysis, of the expression of surface markers on loaded mature dendritic cells at the time of injection.

. The median yield of DC from freshly isolated PBMC was 4.8 × 10^6^ per injection (range: 0.8–13 × 10^6^; *n*=37), which was significantly (Mann–Whitney test: *P*=0.0007) less than the median yield of DC from leukapheresis PBMC (median 10, range: 3–18 × 10^6^; *n*=22). The quality of the DC was controlled by the expression of HLA-DR, CD80, CD86 and CD83 (Figure 1

) (Thurner *et al*, 1999).

### Therapy-induced clinical effects

The details of the therapy-induced clinical effects are given in Table 3

**Table 3:** Clinical evolution and outcome

**Patient number**	**Clinical data during vaccination**	**Radiological evolution**	**Therapy-induced clinical effect**	**Follow-up period after surgery (months)**	**Treatment after vaccination, current status**
1	Peritumoral oedema with grade IV neurotoxicity, responding to steroids	Peritumoral oedema from V2; SD during 7–8 weeks, later PD	Yes	7	DOD at 05-2001
2	Nihil	—	Yes	36	CCR
3	Morning stiffness after V5	Transient contrast enhancement after V5	Yes	35	CCR
4	Nihil	Relapse after 3 months	No	12	Surgery, chemotherapy; DOD at 07-2002
5	At moment of V3: thrombocytes=72 000 *μ*l^−1^	Relapse after 3 months	No	23	Temozolomid; Ommaya reservoir and repetitive punctions of cystic fluid; DOD at 11-2003
6	Subdural hygroma after surgery, anaemia after V3: Hb=9 g dl^−1^	CCR of primary tumour; progression and later on regression of metastatic lesion	Yes	7	Abruption of further vaccination; † at 09-2002
7	Nihil	PD after 2 months	No	8	DOD at 10-2002
8	Nihil	Immediate progression	No	4	DOD at 08-2002
9	Night sweating after V4	PD after 2 months	No	7	Chemotherapy; DOD at 12-2002
10	Meningismus after V3	PD after 4 months	No	9	Chemotherapy; DOD at 02-2003
11	Nihil	PD after 16 months	No	17	DOD at 11-2003
12	Nihil	Relapse after 3 months	No	14	Chemotherapy; DOD at 11-2003

CCR=continuous complete remission; DOD=death of disease; Hb=Haemoglobin; PD=progressive disease; SD=stable disease; Vx=vaccine number.

**Figure 2:**
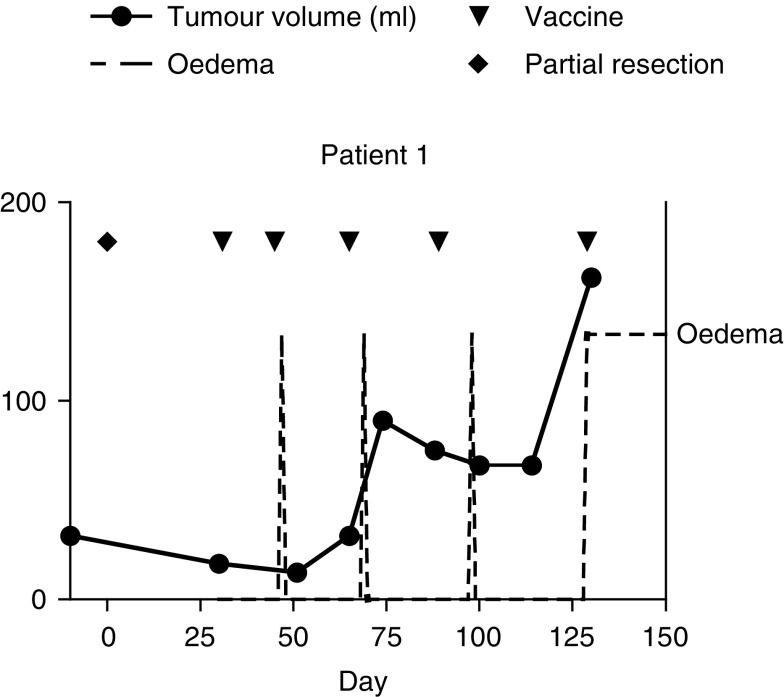
Evolution of tumour volume and response in patient 1. Pre- and postoperative time course of tumour volume (assessed on consecutive MRI images), and of peritumoral oedema reaction (assessed by neurological clinical examination and emergency CT). The time points of operation (day 0) and vaccines (days 31, 45, 65, 89, 129) are indicated.

**Figure 3:**
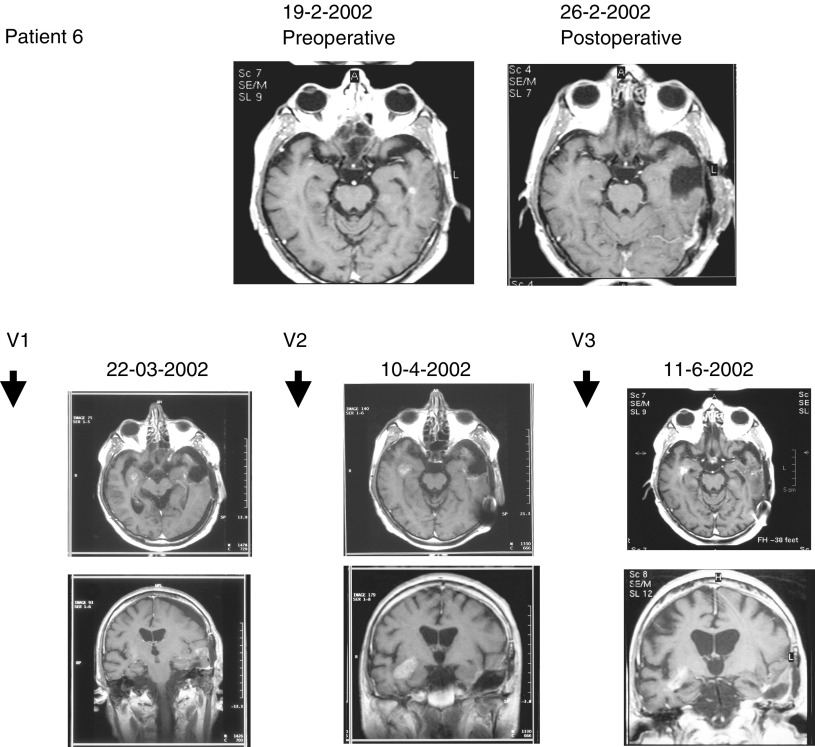
Continuous complete remission of reoperated tumour and partial response of second localisation in patient 6. MRI scan at time of pre- and postoperative status (upper panel); and postoperative evolution on MRI scan during and after vaccination (lower panel). Tumour resection was performed at 25-2-2002. Vaccinations were given at 14-3-02, 28-3-02 and 25-4-02. After the third vaccination, tumour volume in the right temporal lobe decreased by 50%.

. The progression free survival (PFS) and overall survival (OS) at 36 months for the total group was 17% (median PFS=3 months; OS=10.5 months). In the six patients with residual tumour load after surgery and prior to vaccination, one stable disease (patient 1, Figure 2

) and one partial response of a metastatic lesion (patient 6, Figure 3

) were observed based on the volumetric analysis of the tumour. In the latter patient, the right temporal lesion, displaying a high grade malignant metabolic uptake ratio of 2.95 on methionine-PET, decreased in volume by 50% after the third vaccination (Figure 3). In two out of six patients (patients 2 and 3) who had complete resection of their tumour, continuous complete remission was observed at the moment of writing the manuscript, with a follow-up of 36 and 35 months respectively. Patient 3 had a transient contrast enhancement around the resection cavity after the fifth vaccine together with a transient increase of metabolic activity around the resection cavity, measured by methionine-PET (De Vleeschouwer *et al*, 2004). Although the patient numbers were small, patient outcome was not depending on the procedure of making DC or on the amount of proteins to load DC or on the total amount of DC injected. Overall, in four out of 12 patients (patients 1, 2, 3 and 6), some evidence for tumour control due to immunotherapy was observed, and in three of them (patient 1, 3 and 6) an objective response was measured.

### Immune response

In eight patients, a DTH skin test with crude autologous tumour homogenate was performed, of which only two tests remained negative at the time of third vaccination. In one patient, the skin test at the time of the second vaccination was negative, but became positive when the test was performed again at the time of the sixth vaccination. In patient 1, the peritumoral oedema related to each vaccination since the second vaccination was considered as immune-mediated. Therefore, no additional skin test was performed in parallel. Due to the small amount of tumour proteins available in the other patients, the skin test could not be carried out. No relation between positive skin test and response of the tumour or survival of the patient could be found in this small group of patients. There was also no trend in any direction that the amount of proteins used to load DC or the amount of DC injected had any effect on the induction of DTH reaction or on the clinical outcome.

### Toxicity

No severe adverse events were noted, except for one patient. This patient suffered from peritumoral oedema that caused grade IV (NCI common toxicity criteria) neurological deficits and lethargy after vaccinations 2–5, but not after the first vaccination. Remarkably, the period of oedema appeared 30 h after V2, 4 days after V3, and 9 days after V4, while the tumour volume calculated according to the formula *A* × *B* × *C*/2 (*A*, *B* and *C* are the largest diameters in axial, sagittal and coronal plane) remained stable. These symptoms were successfully controlled within 48 h by administering steroids. The other 11 patients did not require corticosteroids during the vaccination period.

In patient 3, a transient contrast enhancement was accompanied by a transient history of more pronounced morning stiffness (De Vleeschouwer *et al*, 2004). Further toxicities were grade II hemato-toxicity in one blood sample (patients 5 and 6), nocturnal sweating after the fourth vaccine (patient 9), and meningeal irritation after the third vaccine (patient 10). In the latter patient, lumbar puncture at that time revealed 8.4 white blood cells per *μ*l with 83% lymphocytes. The CSF was haemorrhagic, with proteins of 1511 mg l^−1^, glucose of 50 mg dl^−1^ and lactate of 2.92 mmol l^−1^. Bacterial cultures and viral PCR tests remained negative. In the other patients, no vaccine-related toxicity was observed.

Only patient 1 and patient 10 have been admitted into hospital due to vaccine-related symptoms. All the other patients received their treatment as outpatients. In patient 8, tumour progression after the partial resection was so overwhelming that only two vaccines could be administered because of the rapid decline of the patient's neurological status.

## DISCUSSION

We summarised the observations on 12 patients with relapsed malignant glioma who were vaccinated with autologous DC loaded with autologous crude tumour homogenate after reoperation. In 25% of patients, we documented an objective clinical response. The report illustrates that, in spite of considerable logistical and technical difficulties, it is worthwhile to further develop the approach of protein-loaded DC as therapy against malignant glioma, even in the absence of known target antigens and for tumours in immunologically privileged sites such as the brain.

An increasing number of clinical trials evaluate DC-based vaccines in the therapy of cancer in adult (Jefford *et al*, 2001) and in pediatric patients (Geiger *et al*, 2001). Specific peptides were mostly used in DC vaccination strategies for melanoma, prostate cancer or breast/ovarian cancer. Also for malignant brain tumour, MHC class I-associated peptides have been eluted from cultured autologous glioma cells, and a mixture of peptides was used to load DC since no specific tumour antigenic targets are known (Yu *et al*, 2001). In other trials, tumour proteins instead of peptides have been used to load DC (Hsu *et al*, 1996; Schott *et al*, 2000; Geiger *et al*, 2001; Chang *et al*, 2002; Höltl *et al*, 2002; Wheeler *et al*, 2003; Yamanaka *et al*, 2003; De Vleeschouwer *et al*, 2004). The use of proteins from autologous tumours instead of peptides is now generally considered a valuable approach, certainly when tumour-specific epitopes are not known (Curiel and Curiel, 2002).

The technical aspects of DC vaccination have recently been reviewed (Schuler *et al*, 2003). Intradermal administration of loaded mature DC seems to be preferable. Up to now, only empirical DC schedules are used. Our observational study pointed to some further practical issues according to feasibility. The size of the tumour sample and the yield of tumour proteins available to load DC were different for each patient. Based on laboratory data on antigen-presenting capacity and quality of DC (manuscript submitted), the range to load one million DC was kept between 30 and 200 *μ*g of tumour proteins. Similarly, the number of DC per injection was different for each preparation and reflect an unavoidable heterogeneity commonly encountered in such studies (Geiger *et al*, 2001; Höltl *et al*, 2002). The fact that some or our patients had tumour control obtained after injection of lower numbers of DC is remarkable.

As this is a feasibility study, it is important to stress that the only selection of candidates for the adjuvant DC-based vaccination therapy was the surgical operability: all patients in whom an intended extensive tumour debulking was deemed feasible and safe by the neurosurgeon were eligible. The actual fraction of patients with a recurrent glioma, who possibly could benefit from this adjuvant therapy in this stage, can only be estimated and probably approaches 10%. Not a single included patient, however, dropped out after surgery: only in patient 2 (partial resection), we stopped after the second vaccination because of overwhelming tumour progression with rapid decline of her neurological status.

Immune monitoring was performed with skin tests, when enough tumour material was available. The DTH testing to antigen is one clinical monitoring tool to indicate cellular immunity, although it remains controversial whether or not DTH to autologous tumour can be a reliable correlate of clinical responses (Clay *et al*, 2001). DTH tests are commonly used if a mixture of undefined tumour proteins is used as a source of antigen for the DC (Geiger *et al*, 2001; Yamanaka *et al*, 2003). Additional immune monitoring with Elispot should be implemented when future patients are treated (Geiger *et al*, 2001; Chang *et al*, 2002; Yamanaka *et al*, 2003).

The group of patients was heterogeneous, because we wanted to assess general feasibility of tumour vaccination. Based on the safety (patient 1) and efficacy (patients 2 and 3) data obtained, (sub)total resection of the tumour should be the major inclusion criteria for upcoming DC vaccination strategies. The induction of serious and clinically important peritumoral oedema in our first patient shows a potential and unacceptable vaccination-related risk for patients with partially resected tumours. From an immunological point of view, tumour-induced immune suppressive mechanisms are limited when the tumour burden is lowered (Holladay *et al*, 1994). In addition, steroids can generally be weaned faster in case of (sub)total resection. Previous unpublished studies of our group showed that sufficient numbers of high quality DC cannot be obtained in glioma patients receiving steroids shortly prior to blood sampling. In fact, our observations illustrate in clinical practice that surgery-induced minimal residual disease is a prerequisite for a clinically relevant efficacy of DC vaccination (Smyth *et al*, 2001). Vaccination might induce better survivals in younger patients, in whom complete resection of malignant glioma can be reached more frequently. Moreover, the cytogenetic entity of malignant glioma at younger age differs from adult malignant glioma (Kraus *et al*, 2002) and might also be responsible for different immunological targeting, besides the differences in immune competence at younger age (Wheeler *et al*, 2003). To further implement these issues, the ongoing HGG-IMMUNO-2003 trial of our group includes patients below the age of 60 years with at least subtotal resection of the relapsed tumour. The amount of tumour proteins available should be enough to be able to provide at least five vaccines with at least 5 × 10^6^ DC loaded with at least 50 *μ*g tumour proteins per 10^6^ DC. The clinical effects will be evaluated by determining the PFS, OS and quality of life in a larger series, and in comparison to a matched historical control group. The immunological effects of DC vaccination in these patients will be further elucidated including Elispot immune monitoring.

After standard treatment for newly diagnosed malignant glioma, patients with early relapse and at least subtotally resectable tumours may particularly benefit from adjuvant immunotherapy. DC immunotherapy appears promising as an approach to successfully induce an antitumour immune response and long-lasting tumour control. This may prolong survival of patients with malignant brain tumours without compromising their quality of life.
